# Complete genome sequence of *Opitutales* bacterium strain ASA1, isolated from soil

**DOI:** 10.1128/mra.01032-23

**Published:** 2024-02-08

**Authors:** Md. Samiul Islam, Kyosuke Yamamoto, Naoki Morita, Isao Yumoto, Souichiro Kato, Ryosuke Nakai, Kensuke Igarashi

**Affiliations:** 1 Graduate School of Agriculture, Hokkaido University, Sapporo, Hokkaido, Japan; 2 Bioproduction Research Institute, National Institute of Advanced Industrial Science and Technology (AIST), Sapporo, Hokkaido, Japan; SUNY College of Environmental Science and Forestry, Syracuse, New York, United States

**Keywords:** bacteria, genome, *Verrucomicrobiota*

## Abstract

We present the complete genome of *Opitutales* bacterium ASA1, isolated from soil. The genome is 5,821,695 bp with 4,638 protein-coding sequences. The genome data suggest that this strain belongs to the class *Opitutae* of the phylum *Verrucomicrobiota*, and its genome has six unique biosynthetic gene clusters associated with secondary metabolites.

## ANNOUNCEMENT

The class *Opitutae* of the phylum *Verrucomicrobiota* consists of physiologically and ecologically diverse members, including some soil isolates with ultra-small cell-size characteristics (1–3). Here, we present the complete genome sequence of *Opitutales* strain ASA1.

The microbial sample was collected 5–15 cm below the surface of garden soil at the AIST Hokkaido Center (43.0201 N 141.4182 E). Soil samples were suspended in sterilized saline (0.9% NaCl) and diluted in a 10-fold series. Aliquots (100 µL) from each dilution were inoculated onto 100-fold diluted PYG (containing peptone, yeast extract, and glucose) medium prepared with autoclaving phosphate and agar separately (pH 8.5) (4) and incubated at 25°C in the dark. After 14 days of incubation, small colonies were picked up and transferred to fresh agar plates for pure isolation. The 16S rRNA gene of the resulting strain ASA1 was PCR-amplified using two universal primers, 27F and 1492R, and its partial sequencing was conducted with the universal primer 907R.

For genome sequencing, strain ASA1 was cultured (pH 8.3) in R2A broth (DAIGO; Nihon Pharmaceutical, Tokyo) at 30°C for 2 weeks. DNA extraction was carried out using a phenol-chloroform method (5). After DNA shearing to approximately 10- to 20-kbp fragments using g-TUBE (Covaris), a sequencing library was prepared using a SMRTbell gDNA Sample Amplification Kit (PacBio) and SMRTbell Express Template Prep Kit v.2.0 (PacBio). The library was sequenced with Sequel IIe (PacBio), and then HiFi reads were obtained using SMRT Link v.12.0.0.177059. Note that the default settings were used for all software for subsequent data processes unless otherwise specified. PCR adapters, PCR duplicates, and ≤1,000-bp short reads were removed with lima verson 2.7.1, pbmarkdup v.1.0.3, and Filtlong v.0.2.1 (https://github.com/rrwick/Filtlong, with the options “--min_length 1000” and “--keep_percent 90”), respectively. Finally, 45,742 qualified HiFi reads with an average length of 6,486 bp were obtained (the total sequence length was 296,678,282 bp). The obtained reads were assembled with Flye v.2.9.1-b1780 using the “--pacbio-hifi” mode and the option “-i 3” (6), and then the assembly graphs for assessing completeness was visualized using Bandage v.0.8.1 (7). The assembled circular genome was annotated with DFAST v.1.2.11 (8). To identify the full-length 16S rRNA gene sequence, we conducted a BLASTn search against the National Center for Biotechnology Information nt/nr database (accessed on 12 September 2023). We also calculated the average nucleotide identity of strain ASA1 in comparison to the type strain database using DFAST. Additionally, we employed the antiSMASH v.7.0 online tool to predict biosynthetic gene clusters (9).

The genome consists of a single chromosome of 5,821,695 bp with a 65.6% G + C content. The chromosome contains a total of 4,690 genes, including 4,638 protein-coding genes, 3 rRNA genes, 48 tRNA genes, and 1 CRISPR gene. Strain ASA1 is affiliated with the class *Opitutae* (*Opitutia* corrig.) of the phylum *Verrucomicrobiota*. However, it exhibits low 16S rRNA gene sequence identities (<93%) to the closest type strain, *Alterococcus agarolyticus* (accession no. NR_036763.1), and an undescribed strain, *Opitutus* sp. strain CFH (KY039339.1). Furthermore, the antiSMASH analysis identified six unique biosynthetic gene clusters in the ASA1 genome (Table 1).

**TABLE 1:** Summary of predicted BGCs in the *Opitutales* bacterium strain ASA1 genome by antiSMASH v.7.0^a^

Genomic region	BGC type	Gene position		Most similar known cluster	Most similar type in MIBiG comparison (similarity score, %)
		From	To		
Region 1	NRPS	3,252,427	3,293,703	NA	NRP + Alkaloid from *Pseudomonas fluorescens* (0.31)
Region 2	NRPS	3,572,624	3,618,738	NA	NRP + Alkaloid from *Pseudomonas fluorescens* (0.31)
Region 3	T3PKS	3,629,430	3,670,473	NA	Polyketide from *Candidatus* Entotheonella serta (0.32)
Region 4	Arylpolyene	5,402,678	5,443,805	NA	Other from *Planctomycetales* bacterium 10988 (0.33)
Region 5	NRPS-like	5,540,659	5,581,974	NA	NRP + Saccharide from *Streptomyces hygroscopicus* (0.34)
Region 6	T1PKS and NRPS	5,776,738	5,821,695	NA	NRP from *Pseudomonas* sp. J465 (0.67)

^a^ BGC, biosynthetic gene cluster; NRP, nonribosomal peptide; NRPS, nonribosomal peptide synthase; T3PKS, Type III polyketide synthase.

## Data Availability

The ASA1 genome sequence was deposited in the DDBJ/ENA/GenBank database under accession number AP028972.1 (BioProject/BioSample PRJDB16742/SAMD00647589; DDBJ Sequence Read Archive DRA017385).
